# What Genetic Modifications of Source Pigs Are Essential and Sufficient for Cell, Tissue, and Organ Xenotransplantation?

**DOI:** 10.3389/ti.2024.13681

**Published:** 2024-12-04

**Authors:** Asghar Ali, Mayuko Kurome, Barbara Kessler, Elisabeth Kemter, Eckhard Wolf

**Affiliations:** ^1^ Molecular Animal Breeding and Biotechnology, Gene Center and Department of Veterinary Sciences, LMU Munich, Munich, Germany; ^2^ Center for Innovative Medical Models (CiMM), LMU Munich, Oberschleißheim, Germany; ^3^ Interfaculty Center for Endocrine and Cardiovascular Disease Network Modelling and Clinical Transfer (ICONLMU), LMU Munich, Munich, Germany; ^4^ German Center for Diabetes Research (DZD), Neuherberg, Germany

**Keywords:** pig, genetic modification, xeno-antigens, complement activation, coagulation dysregulation

## Abstract

Xenotransplantation of porcine organs has made remarkable progress towards clinical application. A key factor has been the generation of genetically multi-modified source pigs for xenotransplants, protected against immune rejection and coagulation dysregulation. While efficient gene editing tools and multi-cistronic expression cassettes facilitate sophisticated and complex genetic modifications with multiple gene knockouts and protective transgenes, an increasing number of independently segregating genetic units complicates the breeding of the source pigs. Therefore, an optimal combination of essential genetic modifications may be preferable to extensive editing of the source pigs. Here, we discuss the prioritization of genetic modifications to achieve long-term survival and function of xenotransplants and summarise the genotypes that have been most successful for xenogeneic heart, kidney, and islet transplantation. Specific emphasis is given to the choice of the breed/genetic background of the source pigs. Moreover, multimodal deep phenotyping of porcine organs after xenotransplantation into human decedents will be discussed as a strategy for selecting essential genetic modifications of the source pigs. In addition to germ-line gene editing, some of these modifications may also be induced during organ preservation/perfusion, as demonstrated recently by the successful knockdown of swine leukocyte antigens in porcine lungs during *ex vivo* perfusion.

## Introduction

Despite their large phylogenetic distance from humans, pigs have become the preferred species as a source of cells, tissues, and organs for xenotransplantation. Major reasons include their favorable reproductive biology (a gestation period of less than 4 months, multiple offspring, sexual maturity at 5–8 months), their propagation in designated pathogen-free (DPF) facilities, the ethically accepted use of pigs for medical purposes, and an established toolkit for efficient and precise genetic modification [1]. The latter is necessary because pigs are a discordant organ source for humans in several respects. Most importantly, porcine cells carry carbohydrate antigens, such as galactose-α1,3-galactose (αGal), N-glycolylneuraminic acid (Neu5Gc), and an Sda-like blood group antigen, against which humans and, in part, also non-human primates (NHPs) have pre-formed natural antibodies (pnAbs). Upon xenotransplantation of porcine tissues or organs into primates, the pnAbs bind to their carbohydrate antigen targets, inducing activation of the complement system, endothelial cell activation, coagulation, and antibody-dependent cellular cytotoxicity (ADCC). Therefore, wild-type porcine xenotransplants are hyperacutely rejected upon transplantation into humans and non-human primates [2].

This can be addressed by generating source pigs lacking the major carbohydrate antigens and expressing one or several human complement pathway regulatory proteins (see below). However, coagulation dysregulation can still occur after xenogeneic organ transplantation due to incompatibilities between membrane-bound factors on porcine endothelial cells and soluble components in human/NHP blood [3]. An example is the thrombin-thrombomodulin (THBD) interaction. Within a species, THBD on the endothelial cells binds thrombin from the circulation, and – supported by an endothelial protein C receptor (EPCR) – the THBD-thrombin complex activates protein C. Activated protein C has an anti-coagulation effect. In a pig-to-primate organ xenotransplant, the porcine endothelium is exposed to human/NHP blood. Porcine THBD can bind human/NHP thrombin, but the complex is inefficient in the activation of protein C, leading to coagulation in the small blood vessels and thrombotic microangiopathy. Thus, adaptations of the source pigs are mandatory to avoid coagulation dysregulation in xenotransplanted organs (see below).

**FIGURE 1:**
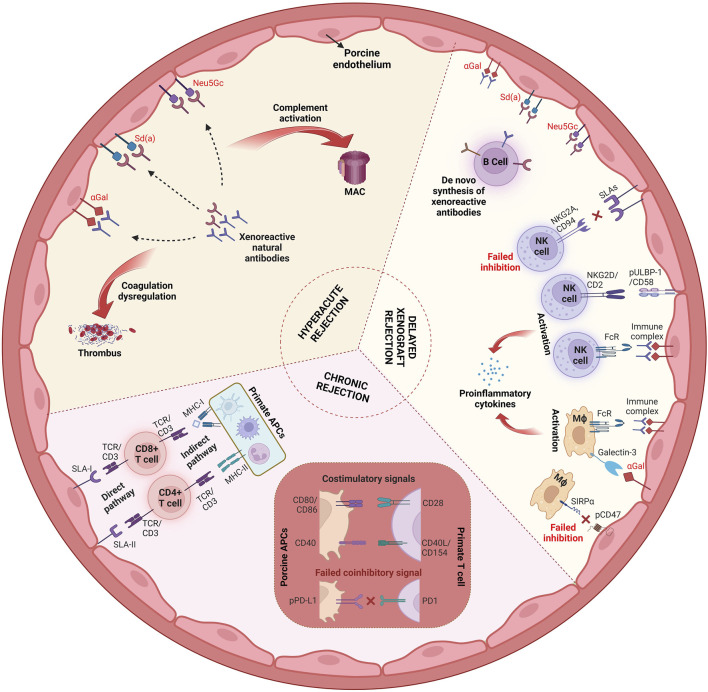
Mechanisms of xenograft rejection. Abbreviations: MAC, membrane attack complex; NKG2A, inhibitory NK receptor; NKG2D, activating NK receptor; pULBP-1, porcine UL-16-binding protein; FcR, Fc receptor; SLA, swine leukocyte antigen; Mϕ, macrophage; PD1, programmed cell death protein 1; pPD-L1, porcine programmed death ligand-1; APC, antigen-presenting cell; MHC, major histocompatibility complex; TCR, T cell receptor (Created with BioRender.com).

In addition, porcine xenotransplants undergo cell-mediated rejection as they trigger, or fail to prevent, the activation of innate immune cells, such as natural killer (NK) cells and macrophages, as well as the adaptive cellular immune system (T and B cells). As swine leukocyte antigen (SLA)-I cannot effectively bind primate inhibitory NK cell receptors, there is, in addition to ADCC, direct human NK cell cytotoxicity against porcine cells. Moreover, macrophages are activated by porcine cells because porcine CD47 does not bind the “do not eat me” signal regulatory protein alpha (SIRPα) on human macrophages. Activation of human or NHP T cells against porcine xenotransplants occurs either directly via the presentation of porcine peptides by porcine antigen-presenting cells (APCs) or indirectly via human or NHP APCs. Several costimulatory [most importantly CD40—CD40L (CD154) and CD80/86—CD28] and coinhibitory signals (PD-L1—PD-1) are involved in this process. B cells contribute to xenograft rejection by producing antibodies that target the graft, leading to complement activation, ADCC, and chronic immune responses that result in graft rejection. Figure 1 provides an overview of the mechanisms involved in xenograft rejection.

## Genetic Modification of Source Pigs for Cell, Tissue, and Organ Transplantation

**FIGURE 2:**
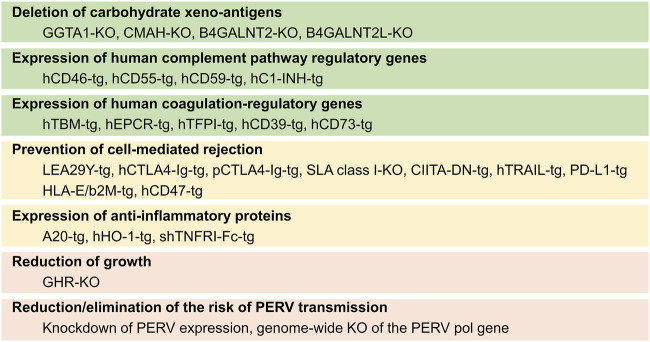
Summary of genetic modifications in source pigs for xenotransplantation and the goals for which they are introduced. KO, knockout/inactivation; tg, transgenic.

The use of genetically modified (GM) source pigs for cell, tissue, and organ transplantation has been extensively reviewed recently [4, 5]. The most prominent modifications, in particular, those tested in transplantation studies in NHPs or even human brain-dead or live patients are shown in Figure 2.

There is broad consensus that the elimination of the primary xeno-antigens (αGal, Neu5Gc, and Sda) is mandatory to prevent the binding of preformed natural primate anti-pig antibodies. This is achieved by introducing loss-of-function mutations of the α-1,3-galactosyltransferase (*GGTA1*), cytidine monophosphate-N-acetylneuraminic acid hydroxylase (*CMAH*), and β-1,4-N-acetyl-galactosaminyl transferase 2 (*B4GALNT2*)/B4GALNT2-like (*B4GALNT2L*) genes, thus producing triple knockout (TKO) pigs. To minimize complement-mediated injury to the xenotransplant, which can also be triggered by ischemia-reperfusion injury or by the binding of antibodies produced *de novo* against porcine antigens, the transgenic expression of one or several human complement pathway regulatory proteins (CPRPs), such as CD46, CD55, and CD59, is often attempted (Figure 2).

Coagulation dysregulation has been overcome by transgenic expression of human THBD in the source pigs. While porcine EPCR appears to be functionally compatible with the human protein C pathway, transgenic overexpression of human EPCR is expected to enhance protective thromboregulation. Other genetic modifications targeting coagulation dysregulation include transgenes for human tissue factor (TF) pathway inhibitor (TFPI), human ectonucleoside triphosphate diphosphohydrolase 1 (CD39), 5′-nucleotidase ecto (NT5E, CD73), or the siRNA-mediated knockdown of porcine TF expression.

Transgenic expression of human leukocyte antigen (HLA)-E/beta2-microglobulin (B2M) is a strategy to inhibit the activation of human NK cells carrying the inhibitory receptor CD94/NKG2A, while the activation of human macrophages can be inhibited by the expression of human CD47. Transgenes for CTLA4-Ig or its higher-affinity derivative LEA29Y have been developed to block the CD28—CD80/CD86 co-stimulatory pathway of T cell activation. Alternatively, the negative coregulatory PD-L1—PD-1 pathway was employed by transgenic expression of human PD-L1. Pigs lacking or expressing reduced levels of SLA class I have been achieved by knockout or knockdown strategies of B2M, while the expression of SLA class II was reduced by transgenic expression of a human dominant-negative mutant class II transactivator (CIITA-DN).

In addition, transgenic pigs expressing anti-inflammatory proteins such as human TNF-alpha-induced protein 3 (TNFAIP3 alias A20), human heme oxygenase 1 (HMOX1), or soluble human tumor necrosis factor receptor I IgG (1)-Fc (shTNFRI-Fc) have been produced to diminish inflammation escaping control by other genetic modifications (reviewed in [3]).

As a measure to prevent excessive growth of the source pigs and their organs, the knockout of the growth hormone receptor (*GHR*) gene has been proposed [6]. This modification reduces the size of the pigs to about 50% of the wild type, but also causes metabolic changes and leads to obesity [7, 8]. Therefore, it is preferable to use an originally smaller genetic background that fits the size of human recipients. Examples are Yucatan miniature pigs or Auckland Island pigs. The latter are characterized by a high level of genetic homogeneity without signs of inbreeding depression, including a uniform SLA makeup, which facilitates tolerance induction strategies. Moreover, Auckland Island pigs have excellent heart function and appear free of cardiac malformations [9], while Yucatan miniature pigs display an increased rate of ventricular septum defects [10, 11].

Modifications aiming to reduce or eliminate the risk of porcine endogenous retrovirus (PERV) transmission include the knockdown of PERV expression or the genome-wide mutagenesis of the PERV *pol* gene by CRISPR/Cas9 genome editing [12]. Alternative strategies include antiviral treatment and vaccination of the recipient against PERV [13].

## GM Combinations for Xenogeneic Heart Transplantation

As of July 2024, around 1,034 patients were on the waiting list for a heart transplant within the Eurotransplant region, with the global numbers being much higher [14]. We recently published a comprehensive review on the use of GM pigs as donors in cardiac xenotransplantation [15]. Key factors for achieving long-term survival include protecting the xenograft from the host immune response, ensuring nonischemic preservation of the xenograft before implantation, developing a clinically applicable immunosuppressive regimen, and managing post-implantation xenograft growth [15]. By knocking out (KO) the *GGTA1*, *CMAH*, and *B4GALNT2/B4GALNT2L* genes to eliminate xenoantigens in donor pigs, and introducing transgenic expression of human complement and/or coagulation regulatory factors, the host immune response is significantly reduced [16].

**FIGURE 3:**
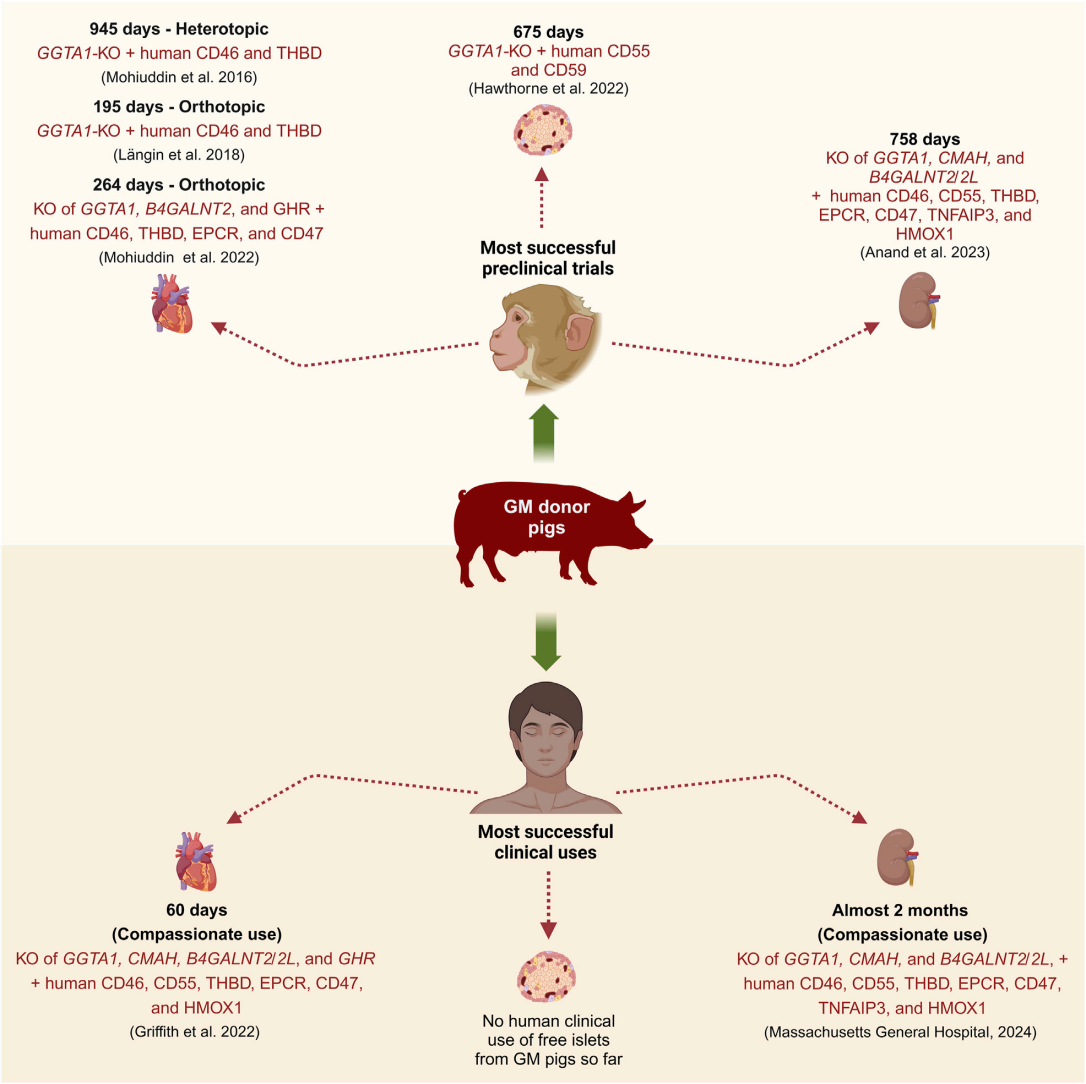
Most successful preclinical and clinical transplantations of GM pig organs or tissue (Created with BioRender.com).

Numerous pig-to-NHP preclinical trials have been conducted to determine the optimal combination of genetic modifications for successful cardiac xenotransplantation [4]. In 2014, Mohiuddin et al. heterotopically transplanted a 3-GM pig heart with *GGTA1*-KO and transgenic expression of human CD46 and THBD into an immunosuppressed baboon and achieved a long-term survival of over 200 days [17]. Building on this success, in 2016, they transplanted in the same heterotopic setting similar 3-GM pig hearts into baboons along with a modified immunosuppressive regimen and achieved survival of up to 2.5 years (Figure 3) [18]. Recently, Chaban et al. [19] performed heterotopic transplantations of hearts from 3-GM (*GGTA1*-KO, *B4GALNT2*-KO, and human CD55 expression), 9-GM (KO of *GGTA1*, *B4GALNT2*, and GHR, along with human CD46, CD55, THBD, EPCR, CD47, and HMOX1 expression), or 10-GM (9-GM plus *CMAH*-KO) donor pigs into immunosuppressed baboons. In this study, a maximum survival time of 393 days was achieved in a 9-GM pig heart recipient [19]. In 2018, the first successful series of life-supporting orthotopic pig-to-NHP heart transplantations was achieved by Längin et al. [20]. They transplanted 3-GM pig hearts with *GGTA1*-KO and transgenic expression of human CD46 and THBD into baboons under standard immunosuppression and achieved a maximum survival of 195 days (Figure 3) [20]. Importantly, in these preclinical trials, post-implantation heart growth was regulated by lowering the recipient’s blood pressure to match the porcine level, regulating cortisone levels, and using the sirolimus prodrug, temsirolimus, to reduce myocardial hypertrophy [20]. In the same year, Hinrichs et al. introduced a novel approach for managing post-transplant heart growth by KO of the *GHR* gene in donor pigs [7]. This genetic modification was first tested by Goerlich et al. in 2021, who orthotopically transplanted 7-GM pig hearts with KOs of *GGTA1*, *B4GALNT2*, and *GHR*, along with transgenic expression of human CD46, THBD, EPCR, and CD47 into immunosuppressed baboons, and achieved survival of up to 264 days [21]. In 2022, Mohiuddin et al. [22] orthotopically transplanted hearts from pigs with 3–9 genetic modifications into baboons. The longest survival time recorded was 264 days in a baboon that received a 7-GM heart, specifically with the KO of *GGTA1*, *B4GALNT2*, and *GHR*, along with human CD46, THBD, EPCR, and CD47 expression [22].

The first pig-to-human life-supporting heart transplantation took place on 7 January 2022, when a 57-year-old man received a 10-GM pig heart from Revivicor. This heart was from a cloned pig and had KOs of *GGTA1, CMAH, B4GALNT2/B4GALNT2L*, and *GHR*, and carried expression cassettes for human CD46, CD55, THBD, EPCR, CD47, and HMOX1 (Figure 3) [23]. The xenograft functioned normally for 49 days post-transplant before experiencing signs of rejection such as sudden myocardial thickening and xenograft failure, leading to the patient’s death on day 60. It was reported that factors such as antibody-mediated rejection, complement-dependent cytotoxicity, and PCMV transmission from the xenograft might have contributed to the xenograft failure [23]. Later on 20 September 2023, a similar 10-GM heart was transplanted in a 58-year-old man, who lived for nearly 6 weeks post-transplant and died on 30 October 2023 [24]. Although the details about the clinical course of this patient are yet to be published, the heart showed signs of rejection days before his passing [24].

## GM Combinations for Xenogeneic Kidney Transplantation

Approximately 1.2 million deaths each year are linked to end-stage renal disease (ESRD) [25, 26], and kidney transplantation remains the most effective treatment option [27–30]. The number of patients waiting for a kidney transplant is strikingly high, with over 10,000 patients waiting in the Eurotransplant region as of July 2024 [14]. Due to comparable sizes and similar metabolic and physiological functions of human and pig kidneys [31], pig-to-human kidney xenotransplantation can be useful to address the organ shortage. However, genetic modifications in donor pigs are required to circumvent certain immunological barriers in pig-to-human kidney xenotransplantation [12, 32].

Multiple research groups have achieved long-term survival of porcine kidney xenograft recipients in NHP preclinical trials [33–37]. Anand et al. and Ma et al. reported survival of up to 758 days using 10-GM pig kidneys, which were modified to KO *GGTA1*, *CMAH*, and *B4GALNT2/B4GALNT2L*, and express human transgenes such as CD46, CD55, THBD, EPCR, CD47, TNFAIP3, and HMOX1 along with retroviral (PERV) inactivation (RI), hence also claimed as 69-GM pigs (Figure 3) [35, 37]. Similarly, Kim et al. and Adams et al. achieved survival of up to 557 days using double KO (*GGTA1* and *B4GALNT2/B4GALNT2L*) and triple KO (*GGTA1*, *B4GALNT2/B4GALNT2L*, and *SLA-I*) pigs, with or without human CD55 transgene [33, 34, 38]. Eisenson et al. reported a maximum survival time of 337 days, using the above-described 10-GM pigs from Revivicor [39]. The insights gained from these studies have paved the way for clinical trials.

In 2022, Montgomery et al. transplanted thymo-kidneys from *GGTA1*-KO pigs into two brain-dead human recipients, in the presence of native kidneys [40]. The study continued for 54 h and a significant increase in urine production and a reduction in creatinine level was observed. Although the initial analysis did not show any signs of hyperacute or antibody-mediated rejection [40], subsequent multimodal profiling revealed an antibody-mediated reaction, with a preference for the glomerular compartment [41]. Locke et al. performed the first clinical-grade trial and transplanted kidneys from the 10-GM Revivicor pigs into two human decedents who first underwent bilateral native nephrectomy [42–44]. The observation period was 74 h in the first and 7 days in the second recipient. In the first study, prominent thrombotic microangiopathy was observed without evidence of cellular rejection or antibody deposition, accompanied by low urine output and high creatine levels [42]. In the second study, the creatine clearance was improved over time, in the absence of thrombotic microangiopathy and some evidence of antibody-mediated rejection [43, 44].

The first compassionate porcine kidney xenotransplantation was done by transplanting a 69-GM pig kidney (3KO.7TG.RI) in an ESRD patient at Massachusetts General Hospital, who unfortunately died 2 months post-transplant due to cardiac arrest with a functioning kidney (Figure 3) [45]. The second patient at New York University suffered from heart and kidney failure, and received a left ventricular assist device (LVAD) on 4th April 2024, followed by thymo-kidney transplantation from a *GGTA1*-KO pig on 12th April 2024 [46]. The kidney xenograft failed after 6 weeks leading to its removal on 29th May 2024 [46].

## GM Combinations for Xenogeneic Islet Transplantation

For patients with type 1 diabetes, β-cell replacement is a superior therapeutic option over daily insulin injections [47]. Xenotransplantation of pig pancreatic islets is a viable alternative to allotransplantation, as discussed in several reviews [5, 48, 49]. Although the isolation of adult porcine islets (APIs) is challenging, some researchers prefer them over neonatal/fetal porcine islets (NPIs) due to their lower α-Gal expression, greater insulin production capacity, and immediate functionality post-transplantation [50]. In contrast, NPIs are easier to isolate but require functional maturation *in vitro*. Nevertheless, many research groups opt for NPIs from the 1- to 5-day-old pancreas and have developed concepts to improve their *in vitro* maturation [51–54].

Regardless of the islet source, over 50% of islets transplanted into the portal vein, a standard protocol in clinics, are lost due to the instant blood-mediated inflammatory reaction (IBMIR) [55]. This innate immune response is more severe for xenoislets than for alloislets [55, 56]. Therefore, overcoming the host immune response is crucial for successful islet xenotransplantation. In pig-to-NHP preclinical trials, surprisingly, the longest islet survival of over 900 days was achieved using WT APIs [57]. However, the recipients required a clinically unacceptable immunosuppressive regimen, and the engrafted pig islets were rejected once the immunosuppression was withdrawn [57]. Genetically modifying the donor pigs can help circumvent the host immune response. APIs from human CD46-expressing pigs survived for over 396 days in immunosuppressed diabetic cynomolgus monkeys [58], whereas NPIs from *GGTA1*-KO pigs survived for up to 249 days in immunosuppressed diabetic rhesus monkeys [59].

Hawthorne et al. [60] transplanted NPIs from 4-GM pigs with *GGTA1*-KO and transgenic expression of human CD55, CD59, and α1,2-fucosyltransferase (HT), into immunosuppressed non-diabetic baboons. Although these NPIs provoked minimal IBMIR, they were rejected 1 month after transplant due to cell-mediated rejection [60]. Bottino et al. [61] transplanted APIs from either 4-GM pigs (*GGTA1*-KO, and transgenic expression of human CD46, TFPI, and CTLA4-Ig), 5-GM pigs (*GGTA1*-KO, and transgenic expression of human CD46, CD39, TFPI, and CTLA4-Ig), or 1-GM pigs (expressing human CD46) into immunosuppressed diabetic cynomolgus monkeys. APIs from both 4- and 5-GM pigs evaded early IBMIR and survived for over 90 days [61]. Recently, Hawthorne et al. [62] transplanted NPIs from 3-GM pigs (*GGTA1*-KO and transgenic expression of CD55 and CD59) in diabetic baboons under judicious immunosuppression and achieved a survival of up to 675 days (Figure 3). A variable survival time of xenoislets in the preclinical trials suggests that optimal combinations of genetic modifications and clinically acceptable immunosuppression regimens are yet to be found.

Although several clinical trials have been conducted using encapsulated WT or GM porcine islets (reviewed in [5]), the clinical trials using free porcine islets are very limited. In the 1990s, Groth et al. [63] intraportally transplanted WT NPIs into a series of diabetic patients under standard immunosuppression. Although complete insulin independence was not achieved, porcine C-peptide was detectable in the urine of four patients for 200–400 days post-transplantation [63]. In 2011, Wang et al. injected WT NPIs in the hepatic artery of 22 diabetic patients under an immunosuppressive regimen [64]. Daily insulin requirement was reduced in 14 patients, and six patients were followed up for 4–6 years after the transplantation [64].

## Multimodal Deep Phenotyping to Select Essential Genetic Modifications of Source Pigs

A possibility of evidence-based refinement of genetic modification of source pigs for xenotransplantation is the analysis of porcine organs that have been transplanted into brain-dead or live patients. In a recent study, Loupy et al. [41] performed a multimodal phenotyping of two *GGTA1*-KO xeno-kidneys that had been transplanted into two brain-dead human recipients and maintained their circulatory and respiratory activity for 54 h [40]. The complex screening protocol combined histopathology, immunophenotyping (IgM, IgG, C4d, CD68, CD15, NKp46, CD3, CD20, and von Willebrand factor), bulk gene expression profiling, and whole-transcriptome digital spatial profiling, including cell deconvolution, to gain insights into spatially resolved immune reactions to the xenografts after 54 h. The cellular and molecular findings of the study indicated an antibody-mediated reaction preferentially to the glomerular compartment of the xeno-kidneys, associated with endothelial activation and the recruitment and activation of monocytes, macrophages, and natural killer cells, but with no evidence for complement activation. The study also highlighted the potential benefits of GM strategies of source pigs to inhibit the activation of macrophages (such as the expression of human CD47 [65]) and natural killer cells (e.g., expression of HLA-E/B2M [66]). The complex multimodal screening approach as introduced by Loupy et al. [41] has the potential to unravel hitherto unknown reactions to xenotransplants and has recently also been applied to two cases of cardiac xenografts from 10-GM pigs with KOs of *GGTA1*, *CMAH*, *B4GALNT2/B4GALNT2L*, and *GHR* and transgenic expression of human CD46, CD55, CD59, THBD, EPCR, and HMOX1 in brain-dead recipients [67, 68].

In contrast to life-supporting xenogeneic heart transplantation experiments in baboons [20, 69] and the first compassionate use transplantations in live patients [23], perfusion preservation of the porcine hearts was not performed in these studies in decedents [67]. Furthermore, standard immunosuppression plus the complement inhibitor eculizumab, but not CD40-CD154 co-stimulation blockade was used. While in both cases the xeno-heart was fully functional immediately after transplantation, the function declined in one case due to organ size mismatch and associated tissue hypoperfusion. The second heart was better size-matched and performed well throughout the study. The multimodal screening [68] of the hearts revealed corresponding molecular changes: an early immune response driven by T cell and natural killer cell activity and disruptions in cellular metabolism in the first heart, and only relatively minor changes in RNA, protein, lipid, and metabolic profiles in the second case.

Although the conclusions of these multimodal phenotyping studies are limited by the small sample size and short study duration [41, 68], the general strategy may provide new insights into human anti-porcine xeno-organ responses. This approach could also be applied to xenotransplantation studies in nonhuman primates where larger series with long-term outcomes exist [20, 22, 37, 39, 69].

## Genetic Modification of the Organ vs. the Source Pig

An interesting concept is the modification of organs for transplantation during preservation perfusion. Recently, Figueiredo and colleagues [70] explored this concept in a porcine allogeneic lung transplantation model. Donor lungs were lentivirally transduced during *ex vivo* perfusion to express short hairpin RNAs targeting β2-microglobulin (B2M) and class II transactivator mRNAs to knock down SLA expression. While all grafts in the control group with unmodified SLA expression (n = 7) were rejected within 3 months, five of the seven animals in the SLA-knockdown group maintained graft survival without immunosuppression during the 2-year monitoring period, demonstrating a clear survival advantage of the SLA-silenced organs [70]. The reduction of SLAs may be also important for xenotransplantation since late failure of pig-to-rhesus renal xenografts was associated with an increase in anti-swine leukocyte antigen class I and class II, particularly anti-SLA-DQ, antibodies [71].

## Conclusion and Outlook

Defining what constitutes “sufficient genetic modifications for successful xenotransplantation” is one of the most frequently asked questions in the field and is equally challenging to address. Numerous research groups have made significant progress in identifying the key genetic modifications to evade the host immune response and ensure long-term xenograft survival. While various GM combinations have been tested *in vitro* and in preclinical trials, there is a broad consensus on some of the critical genetic modifications. These include the elimination of porcine xenoantigens, and transgenic expression of human proteins to inhibit complement activation and coagulation dysregulation. Notably, a similar combination of genetic modifications, including *GGTA1*-KO, and transgenic expression of human CD46 and THBD, was used in the most successful preclinical trials in both heterotopic and orthotopic cardiac xenotransplantation, resulting in consistent long-term xenograft survival. We consider this combination as “minimum essential genetic modifications.” However, similar success is yet to be demonstrated in clinical trials. More preclinical and clinical trials are needed to determine whether additional genetic modifications, beyond the minimum-essential ones, can synergize to enhance the efficacy of xenotransplantation. To minimize the potential side effects of extensive genetic modifications, as well as off-target effects from gene-editing tools, it is crucial to critically assess the necessity of each additional genetic modification. Some studies advocate for further genetic modifications to address cell-mediated rejection, eliminate PERVs, and regulate post-transplant organ growth. However, if an appropriate pig breed is chosen, certain genetic modifications can be avoided. For instance, Auckland Island pigs have a naturally small body stature and were selected to be free of PERV-C, eliminating the requirement for additional gene edits to regulate the organ size or remove PERVs.

Recently, the compassionate use of 10-GM porcine hearts and kidneys in humans achieved a maximum survival of 2 months. Although this survival time is shorter compared to what was observed in preclinical trials, it marks a significant milestone in the field of xenotransplantation. Importantly, these source pigs had the aforementioned minimum essential genetic modifications as well as some additional ones. However, the published reports from these clinical studies do not provide details on the expression level of human transgenes in the xenografts. We speculate that the level of transgene expression might be more critical compared to the number of transgenes for achieving long-term survival and function of the xenograft. Nevertheless, these cases underscore the importance of continuous innovation in genetic modifications and immunosuppressive strategies for successful xenotransplantation.

The use of multimodal deep phenotyping techniques in xenotransplantation research has provided valuable insights into immune responses and potential improvements for the success of organ transplants from genetically modified pigs. By integrating various analytical approaches, such as histopathology, immunophenotyping, gene expression profiling, and spatial transcriptomics, researchers have been able to identify specific immune reactions and molecular changes associated with xenograft rejection or acceptance. Despite the limitations of small sample sizes and short study durations, the ongoing advancements in genetic engineering of donor pigs, coupled with the insights gained from preclinical and early clinical trials, are paving the way for xenotransplantation to become a viable and life-saving solution for patients in need of organ transplants.
